# The Oleaginous Yeast *Metschnikowia pulcherrima* Displays Killer Activity against Avian-Derived Pathogenic Bacteria

**DOI:** 10.3390/biology10121227

**Published:** 2021-11-24

**Authors:** Robert H. Hicks, Mauro Moreno-Beltrán, Deborah Gore-Lloyd, Christopher J. Chuck, Daniel A. Henk

**Affiliations:** 1 Milner Centre for Evolution, University of Bath, Bath BA2 7AY, UK; robert.hicks@epsrc.ukri.org (R.H.H.); maurom@chalmers.se (M.M.-B.); debslloyd@hotmail.co.uk (D.G.-L.); 2 Department of Chemical Engineering, University of Bath, Bath BA2 7AY, UK; c.chuck@bath.ac.uk

**Keywords:** antimicrobial, fungal–bacterial interaction, poultry disease

## Abstract

**Simple Summary:**

Pathogenic bacteria in poultry and the widespread use of antibiotics to manage them are costly in terms of production, environmental risk and human health. Probiotic and other low-cost, non-antibiotic treatments offer attractive alternatives to antibiotic applications, but relatively few of these options exist. In this research, we investigated the potential of an otherwise-useful industrial yeast, *Metschnikowia pulcherrima*, for the active suppression of poultry pathogenic bacteria. We tested multiple strains of yeast against several important bacterial pathogens and found that the more inhibitory strains of yeast supressed bacterial growth and actively killed the most recalcitrant bacteria. Less aggressive yeast strains could increase the growth of some bacterial strains in some environments. The yeast produced novel molecules in response to the presence of the bacteria and we identified several potential mechanisms by which the yeast inhibited or killed bacteria. Together, these results point towards a useful application of a novel yeast for enhanced, antibiotic-free pathogen control.

**Abstract:**

*Metschnikowia pulcherrima* is a non-conventional yeast with potential to be used in biotechnological processes, especially those involving low-cost feedstock exploitation and biocontrol applications. The combination of traits that supports these industrial applications in *M. pulcherrima* also makes it an attractive option to study in the context of livestock health. In this study, we examined the specific interactions between *M. pulcherrima* and multiple avian pathogenic bacteria. We tested individual bacteria–yeast interactions and bacterial combinations in both solid and liquid media and in variable nutrient environments. Across multiple isolates of *M. pulcherrima*, we observed different levels of antimicrobial activity, varying from supporting the growth of competing bacteria through suppression and bacterial killing, and we found that these responses varied depending on the bacterial strains and media. We identified multiple molecular routes, including proteins produced by *M. pulcherrima* strains, that acted to control these microbial interactions. Furthermore, protein screening revealed that *M. pulcherrima* strains were induced to produce proteins specifically when exposed to bacterial strains, suggesting that fine-tuned mechanisms allow *M. pulcherrima* to function as a potential lynchpin in a microbial community.

## 1. Introduction

The use of antibiotics in animal farming is a global practice. Whether prescribed therapeutically when clinical symptoms appear or prophylactically for growth proportion and where farming conditions or methods predispose a population to disease outbreaks, the benefits of their use are important both financially and from an animal health perspective. Pigs whose diets are supplemented with antibiotics can require substantially less feed to achieve desired growth, while in chickens, egg production and hatchability for broilers is significantly improved [1].

Improved animal health measured by growth and overall productivity results from a variety of mechanisms associated with antibiotic use. Aside from the loss of available nutrients due to the additional bacterial load carried, antibiotics can thin the gut mucus membrane to aid absorption and remove the need for animals to produce defensive cytokines, which can lead to muscle wastage [2]. Furthermore, the maintenance of a healthy commensal microbial population though prophylactic administration benefits animals by aiding the digestion and fermentation of plant polymers, the synthesis of vitamins and the conversion of toxins to non-toxic compounds and by forming an extra line of defence against pathogen colonisation [3]. For these reasons, approximately 80% of food animals receive antibiotic medication for some or most of their lives [4].

Such widespread antibiotic use has correlated with the development of antibiotic-resistant strains, reducing antibiotics’ efficacy in the animals they are prescribed to. The emergence of resistant strains is thought to be accelerated in livestock by the use of prophylactic antibiotic growth-promoting strategies, which involve administering antibiotics in sub-therapeutic dosages. Though an issue for agricultural farming, antibiotic-resistant strains also present a serious public health issue, as many are also pathogenic to humans [5,6]. Indeed, a list compiled by the World Organisation for Animal Health that detailed the “critically important” antibiotics for livestock included representatives of all antibiotic classes used routinely in human medicine [7,8]. Attempts to alleviate the tendency for livestock to act as reservoirs of resistance to essential human therapeutics by substituting specific antibiotics with analogues not used in humans have also failed, yielding tolerance to existing therapeutics [9]. 

Concerns over the impacts of antimicrobial resistance led to regulations being set by the European Union in 2006, restricting European farmers to antibiotic use via veterinary prescription only [10]. In some instances, where appropriate, veterinarians can alternatively prescribe vaccines to eliminate antibiotic use altogether [11]. Though these strict EU regulations have placed pressure for similar compliance on the global market, a mixed response has been observed in different countries. In the US, efforts to reduce antibiotic use by the Food and Drug Association remain on a voluntary basis, whilst restriction commitments in Japan cut antibiotic use by a third between 2000 and 2013, with a further third committed to in 2020 [10]. In China, there are currently no bans on the direct use of antibiotics as growth promoters [12]. Concerns have also bolstered a movement towards antibiotic-free farming as a marketing tool in both pork and chicken production.

Though restrictive action varies by country, the use of low-dose prophylactic antibiotic administration for the purpose of growth promotion is the most problematic with regard to resistance reservoirs. As a result, the practice of adding probiotic microorganisms into animal feed has increased substantially, correlating with increased research into the effect of microbial communities within the gastro-intestinal tract (GIT) on animal productivity [3,13]. Benefits of effective probiotics mimic those of antibiotics: improved health and increased productivity in livestock animals. Probiotic benefits can, however, go further by boosting or improving the existing GIT microbiota, allowing for improved immunity against pathogens and assisting in feed digestion [14]. In poultry, incidences of diseases such as salmonellosis, campylobacteriosis and coccidiosis can be greatly reduced with probiotic treatment, and in pigs, mortality through enterotoxic *E. coli-*caused diarrhoea infections can be reduced. Human health is also positively affected by reduced colonisation of the GIT by *Salmonella* Enteritidis, leading to increased food safety [15]. The ideal probiotic loading is 10^9^ CFU/kg feed, and microorganisms selected for use in the EU are most often Gram-positive bacteria, including commensal species such as *Lactobacillus* and *Enterococcus* as well as non-commensal *Bacillus* species. 

Although bacteria are more commonly used, the inclusion of yeast species in probiotic formulations has increased as research has revealed their positive impacts. Currently, *S. cerevisiae* and *Kluyveromyces* species are most widely used, though species of *Pichia*, *Metschnikowia*, *Candida* and *Yarrowia* have also been reported to have probiotic properties [14,16]. Inclusion of yeast as probiotics may provide additional benefits over those provided by bacteria as they are capable of producing an array of extracellular enzymes active on the cellulose, hemicellulose, starch and proteins within feed, as well as vitamins and trace minerals [16]. Due to rapidly increasing demand, the supplementation of yeast into feed within the poultry sector has gained increased attention. Addition of *S. cerevisiae* into the diets of laying hens improved productivity by both increasing nutrient utilisation and reducing numbers of pathogenic bacterial strains. In one study, 70% of control birds fed with *Salmonella* were subsequently colonised compared to just 20% and 5% for birds fed with 1 g and 100 g of *S. cerevisiae* probiotic per kg of feed, respectively [17]. Reductions in the colonisation by *E. coli* and *Staphylococcus* sp. have also been reported following the addition of live yeast [18]. One hypothesis for the effectiveness of yeasts in limiting bacterial growth is that they manipulate bacterial adherence to the mannose in yeast cell-walls. In this scenario, adhered bacteria do not attach within the GIT and, as yeast have not been demonstrated to permanently colonise within animal hosts, the bacteria and yeast are passed out via excretion [17].

Though there are clear successes with *S. cerevisiae*, investigating alternative yeasts as for use as probiotics could potentially offer further benefits due to differences such as extracellular enzyme production and antimicrobial phenotypes. Though recently the yeast *Metschnikowia pulcherrima* has been utilised as a sustainable palm alternative, prior to this research had focused on its antimicrobial activity [19,20,21,22,23,24]. The inhibitory mechanism is primarily thought to derive from a pH-dependent production of pulcherriminic acid (PA), which chelates iron to form the insoluble red pigment pulcherrimin, thus causing iron depletion in the growth substrate, and this can directly affect cellular pH [22,25]. However, antimicrobial activity was still observed in a mutant *M. pulcherrima* strain that was incapable of producing PA due to a point mutation in *SNF2*, suggesting inhibitory mechanisms beyond PA. Antagonistic activity exhibited by this yeast has led to its inclusion in wine making to prevent spoilage by non-*Saccharomyces* yeasts, as well as its use as a postharvest biocontrol agent on fruits. In this study, *M. pulcherrima* strains were investigated for antimicrobial activity against three avian pathogenic bacteria: *Salmonella*, *Staphylococcus* and avian pathogenic *E. coli* (APEC).

## 2. Materials and Methods

### 2.1. Chemicals

Unless otherwise stated, chemicals were sourced from Sigma-Aldrich (Darmstadt, Germany) and used without further purification.

### 2.2. Strains, Strain Maintenance and Media

Eleven *Metschnikowia pulcherrima* strains were used in this study. DH5, DH10, DH21, ICS1, ICS46, ICS48 and QRI1 were isolated in Bath, UK. NCYC2580 and NCYC3047 were sourced from the National Collection of Yeast Cultures, and 4 × 3 and F3 were strains derived via adaptive evolution of an NCYC2580 progenitor [26,27,28,29]. Strains were maintained on malt extract agar (MEA) plates and re-streaked on a fortnightly basis. Three bacterial species, isolated from avian hosts in Thailand, were used in this study: *Staphylococcus aureus* CN9, *Salmonella enterica* F1Fec3 and avian pathogenic *Escherichia coli* 9002 [30]. Strains were maintained on tryptic soy agar (TSA), pH 5.5, and re-streaked on a fortnightly basis. For the preparation of overnight yeast cultures, a single colony was inoculated into 5 mL Soya Malt Broth (SMB) pH 5 (3% tryptic soy broth, 2.5% malt extract) and incubated at 25 °C with 200 rpm agitation. For overnight bacterial cultures, a single colony was inoculated into 5 mL LB broth–Miller (LB) and incubated at 32 °C with 200 rpm agitation. The yeast nitrogen base (YNB) medium was prepared as follows: 6.7 g L^−1^ yeast nitrogen base, 25 g L^−1^ glucose. Optical densities were measured at 595 nm throughout. 

### 2.3. Zone of Inhibition and Liquid Competition Assays

#### 2.3.1. Zone of Inhibition Assays

Overnight cultures were adjusted to optical densities of 1 and 5 for bacteria and yeast respectively using PBS as the diluent. A total of 200 μL of bacterial suspension was spread onto a TSA plate and, once dry, 3 μL of yeast suspension was spotted on top. Plates were incubated at 25 °C for five days, at which point images and zone-of-inhibition measurements were obtained. A crude proxy for PA production and conversion into pulcherrimin is the size of the red halo surrounding yeast cells when grown on identical TSA plates without bacteria. To mitigate the effect of PA and assay for alternate mechanisms of inhibition, increasing concentrations of 0.01 mM and 0.1 mM FeCl_3_ were supplemented into TSA plates and the ZOI analysis was repeated. 

#### 2.3.2. Liquid Competition Assays

Overnight cultures were adjusted to an optical density of 1 for yeast and bacteria, using PBS as the diluent. A total of 100 μL of each culture dilution was added to 10 mL SMB and incubated at 25 °C with 200 rpm agitation. Bacterial growth was tracked through CFU counting. 

### 2.4. Supernatant Generation for Bacterial Growth Assay and SDS-PAGE

To generate control yeast and bacterial supernatants, overnight cultures were diluted to an optical density of 1 and 100 μL was added to 10 mL of YNB + glucose and grown for 48 h. To generate induced yeast samples, yeast cultures were prepared as before; however, after 24 h a 1 mL suspension of an avian pathogenic *E. coli* (APEC) overnight culture was diluted to OD 5, centrifuged and resuspended in 100 μL PBS, and then the total volume was added to the growing yeast culture. This APEC-supplemented culture was incubated for a further 24 h. After 48 h in total, cultures were centrifuged and the supernatant filter sterilised (0.22 μm). To assess the supernatant effect on the APEC growth rate, an overnight culture of bacteria was diluted to OD 1 and 100 μL was inoculated in 9 mL SMB + 1 mL of each respective filter-sterilised supernatant. Cultures were incubated at 25 °C with 200 rpm agitation, and the optical density of each culture was tracked.

The supernatants of strains Q1 and F3 were assayed for their effects on bacterial growth rate, as well as via crude protein secretome analysis using SDS-PAGE. Supernatants were analysed from yeasts grown separately for 48 h, as well as cultures where APEC was dosed in after 24 h to elicit an induced response and grown for a further 24 h. For SDS-PAGE analysis, 5 mL of filter-sterilised supernatant was precipitated with methanol, centrifuged and allowed to dry. Proteins were resuspended in 1× NuPAGE LDS sample buffer (ThermoFisher, Oslo, Norway) and run at 200 V for 30 min in a 4 to 12% Bis-Tris mini-gel (ThermoFisher, Oslo, Norway). Gels were then stained with SimplyBlue SafeStain (ThermoFisher, Oslo, Norway) and imaged.

### 2.5. Statistical Analyses

Zones of inhibition, halo sizes and OD values were tested for significant differences within experiments using ANOVA followed by Tukey’s HSD. Differences in OD induced by supernatant treatments were assessed using Mann–Whitney tests to compare between pairs of treatments. All statistical tests and boxplots for supernatant treatments were carried out in R version 3.5.3 (R Core Team). Plots for ZOI analysis and competition in liquid culture were created in Microsoft Excel (Microsoft Corporation, Redmond, Washington, DC, USA). 

## 3. Results

### 3.1. ZOI Assays

**Figure 1:**
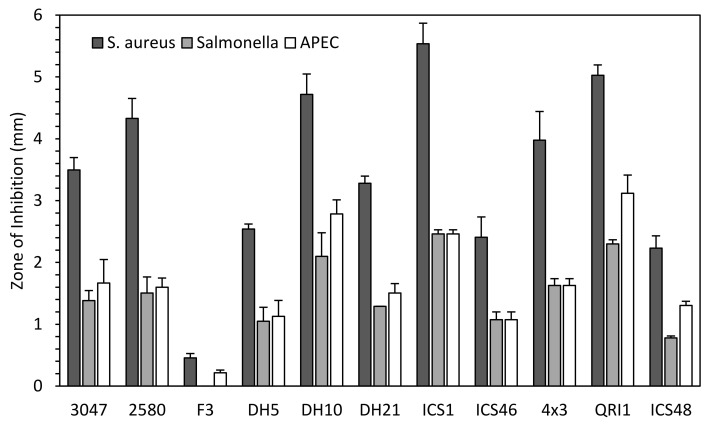
*M. pulcherrima* zone-of-inhibition assay against pathogenic bacteria. A lawn of each bacterium was prepared, onto which an aliquot of yeast was spotted. Plates were grown at 25 °C for five days before zone-of-inhibition measurements were taken. Data represent the values from five plates.

To assess variation in antimicrobial activity across isolates, zone-of-inhibition (ZOI) assays were performed with 11 strains against 3 avian-derived pathogenic bacteria. Overall, *S. aureus* growth was the most inhibited by all yeast strains compared to inhibition of *Salmonella* and avian pathogenic *E. coli* (APEC) (Figure 1). There were minor differences between the ZOI’s against APEC and *Salmonella*, with the latter more resistant to the antimicrobial effects of some strains. A proportionally large difference was observed between yeast strains, with DH10, ICS1 and QRI1 being the most inhibitory strains within this assay and F3 the least. There was no evidence of selectivity in the inhibitory activity of any of the yeasts, meaning that strains were effective against all bacteria tested. 

**Figure 2:**
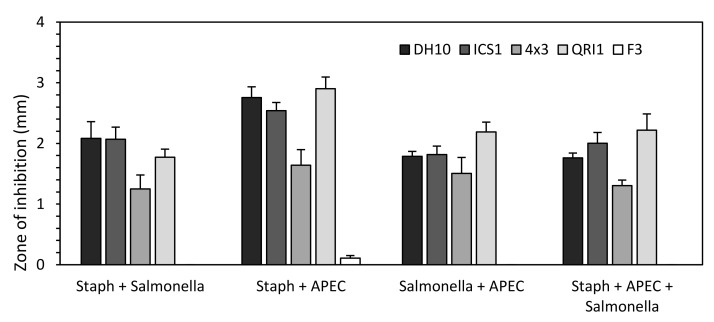
*M. pulcherrima* zone-of-inhibition assay against mixes of pathogenic bacteria. A lawn of each bacterial strain was prepared by spreading a premixed aliquot of bacterial combinations as described, onto which an aliquot of yeast was spotted. Plates were grown at 25 °C for five days before zone-of-inhibition measurements were taken. Data represent the values from five plates.

As pathogenic bacteria are rarely found in monocultures within hosts, the inhibitory effects of selected *M. pulcherrima* strains against bacterial combinations were assessed in the same manner [31]. Five strains were assayed: DH10, ICS1 and QRI1 to represent high inhibitory activity; F3 to represent low inhibitory activity; and 4 × 3, a strain capable of high lipid accumulation. Promisingly, there did not appear to be a synergistic effect of bacterial mixes limiting the ZOI produced by yeast (Figure 2). Rather, the zone of inhibition observed appeared to match the most resistant bacteria; i.e., if the mixture contained *Salmonella*, then the ZOI observed was akin to *Salmonella* alone.

**Figure 3:**
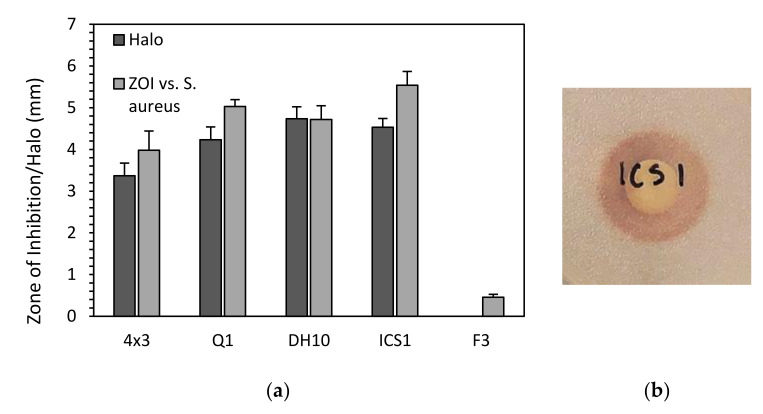
Comparison between pulcherrimin halo and ZOI against *S. aureus.* For halo production, each yeast was spotted onto a TSA plate and grown for five days: (**a**) ZOI vs. *S. aureus* data from Figure 2; (**b**) exemplar halo for strain ICS1.

The halo diffusion for the five selected *M. pulcherimma* strains was assessed by spotting each isolate onto a TSA plate and comparing this with the ZOI against *S. aureus*. Here, a clear correlation was observed between halo size and ZOI, suggesting that the PA mechanism of inhibiting bacterial growth is effective in agar competition assays (Figure 3).

**Figure 4:**
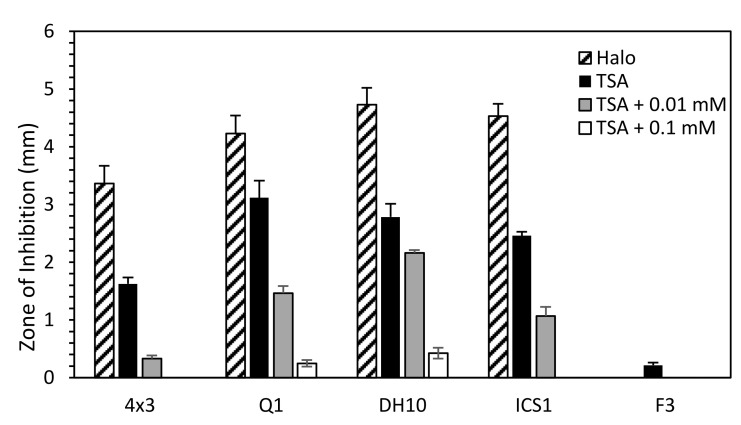
Effect of increasing FeCl_3_ concentration on APEC ZOI. A lawn of APEC was prepared, onto which an aliquot of yeast was spotted. Pates were supplemented with 0, 0.1 and 0.01 mM FeCl_3_ as described. For halo measurement, TSA without additional iron was used. Plates were grown at 25 °C for five days before zone-of-inhibition/halo measurements were taken.

Increasing concentrations of FeCl_3_ reduced inhibition of bacterial growth (Figure 4). Although this trend was true for all *M. pulcherrima* strains, a discrepancy was apparent between ICS1 and the other two high inhibitory strains, QRI1 and DH10. While all three strains had similar halo sizes, ICS1 did not cause an APEC ZOI in a high FeCl_3_ concentration (0.1 mM), but DH10 and QRI1 maintained inhibition, albeit in a reduced form.

### 3.2. Antimicrobial Effects and Competition in Liquid

**Figure 5:**
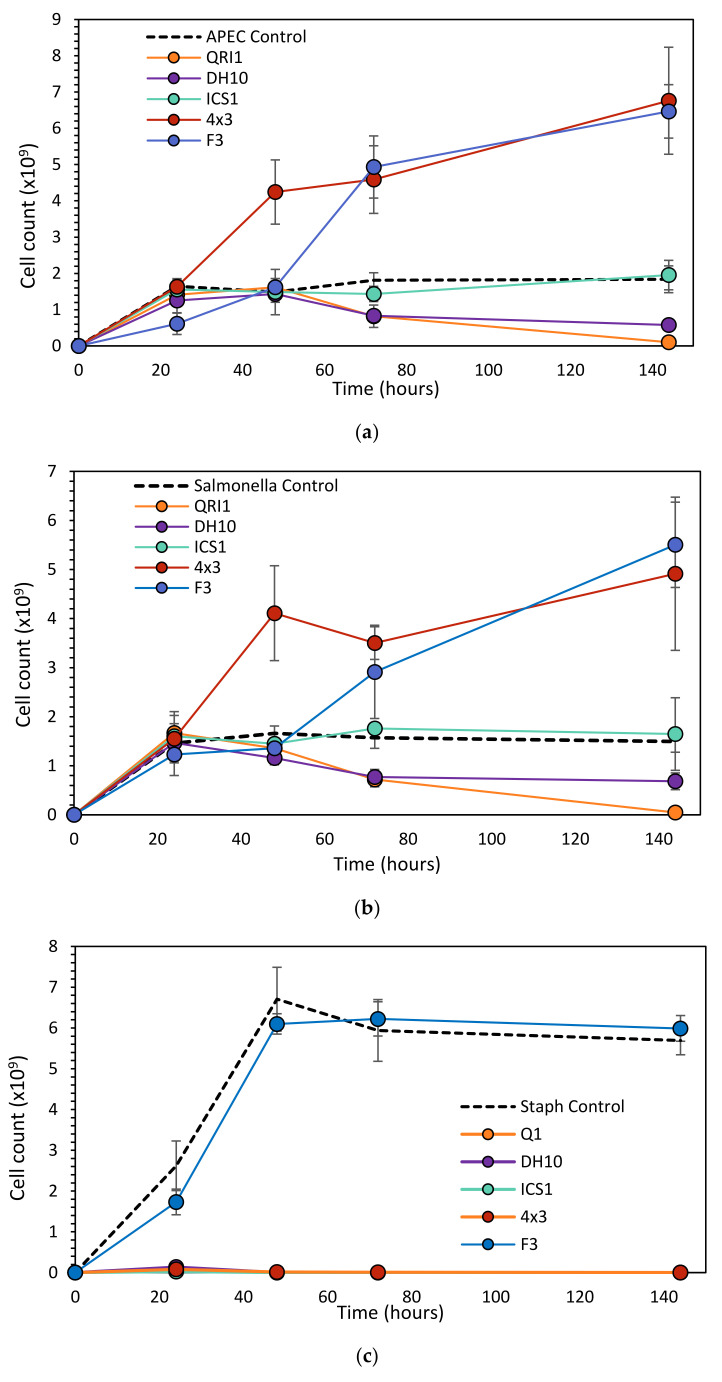
Co-culture of *M. pulcherrima* and pathogenic bacteria in liquid medium. Overnight cultures of bacteria and yeast were diluted to OD 1, and 100 μL of each was inoculated into a 10 mL SMB culture in triplicate. Cultures were incubated at 25 °C, 200 rpm. Bacterial cell counts were measured at 24, 48, 72 and 144 h via serial dilution-plating. Data represent the mean and standard deviation of triplicate cultures: (**a**) APEC co-culture; (**b**) Salmonella co-culture; (**c**) *S. aureus* co-culture.

The antimicrobial effect was most effective against *S. aureus,* for which bacterial counts were reduced to such an extent that they were not detectable via serial dilution-plating (yeast concentrations being too high) for 4 × 3, DH10, ICS1 or QRI1 (Figure 5). F3, on the other hand, did not have any effect on *S. aureus* cell counts relative to the control. When yeast strains were put in competition against APEC and *Salmonella*, 4 × 3 performed poorly in liquid and its presence in co-cultures increased bacterial cell counts relative to the control. Similarly, F3 and *Salmonella*/APEC co-cultures resulted in increased bacterial cell counts relative to the control. For ICS1 and APEC/*Salmonella* co-cultures, bacterial cell counts were comparable to control cultures. Only DH10, and particularly QRI1, were inhibitory within liquid competition. These strains appear to actively reduce bacterial cell counts below their inoculation levels. 

**Figure 6:**
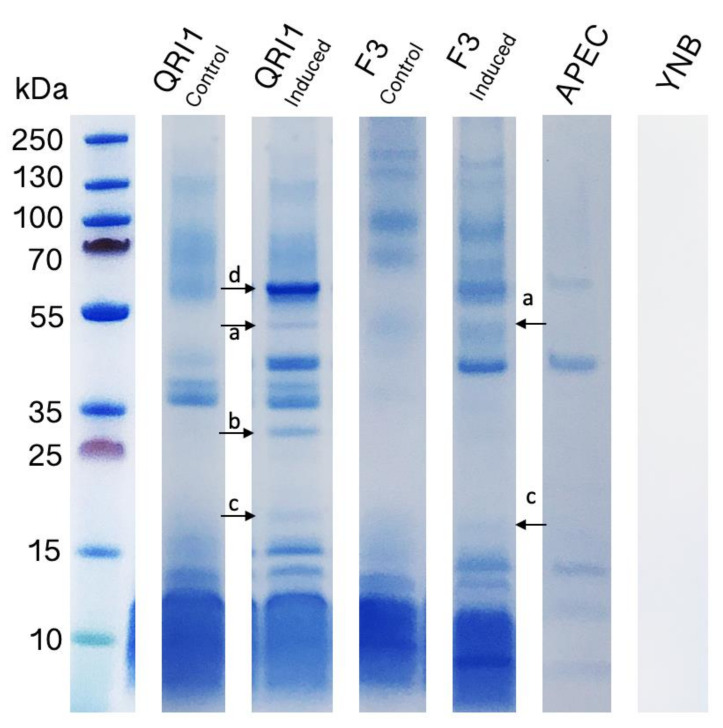
SDS-PAGE of yeast and bacterial supernatants grown in YNB. Control yeast and APEC supernatants were measured after 48 h growth. For induced yeast samples, APEC was added after 24 h and grown for a further 24 h. A total of 5 mL of supernatant was precipitated from each culture. The full Western Blot can be found in Supplementary Materials.

To further understand the non-PA inhibitory mechanisms, the supernatants of high and low inhibitory strains, Q1 and F3, were assayed for their effects on the bacterial growth rate, and crude protein secretome analysis was also undertaken. Significant differences in protein profiles were clearly visible between the control samples of QRI1 and F3 using SDS-PAGE (Figure 6). Though some protein bands were shared, notable differences included two bands >130 kDa present in F3 but not QRI1 and two prominent bands around 40–45 kDa in QRI1 but not F3. Both strains produced a large, indistinguishable collection of proteins around 10 kDa.

In analysing the induced yeast samples, we considered the changes in protein profiles produced by APEC and discounted these as ”new” bands. A band of approximately 55 kDa (band a) appeared in the QRI1-induced sample and not in the control, and it also appeared in the F3-induced sample, though there was a suggestion that it appeared in the control too. A second shared change occurred in a band at approximately 17 kDa (band c) in induced yeast samples, though this was clearly more abundant in QRI1. Interestingly, there was a strong band at approximately 30 kDa (band b) present in only the QRI1-induced sample. It is noticeable that the three most prominent bands in the APEC control sample were stronger in the QRI1-induced sample than in F3 (i.e., band d), suggesting lysis. It is also worth noting that, due to the large group of proteins produced by both yeasts around 10 kDa, proteins which may have been induced by APEC were lost. Despite this, it is clear that the secreted proteomes of QRI1 and F3 were different when grown on identical, minimal media, and this may account for some apparent differences in antimicrobial effects. The analysis also uncovered a protein with potential lytic activity in QRI1, as highlighted in band b.

**Figure 7:**
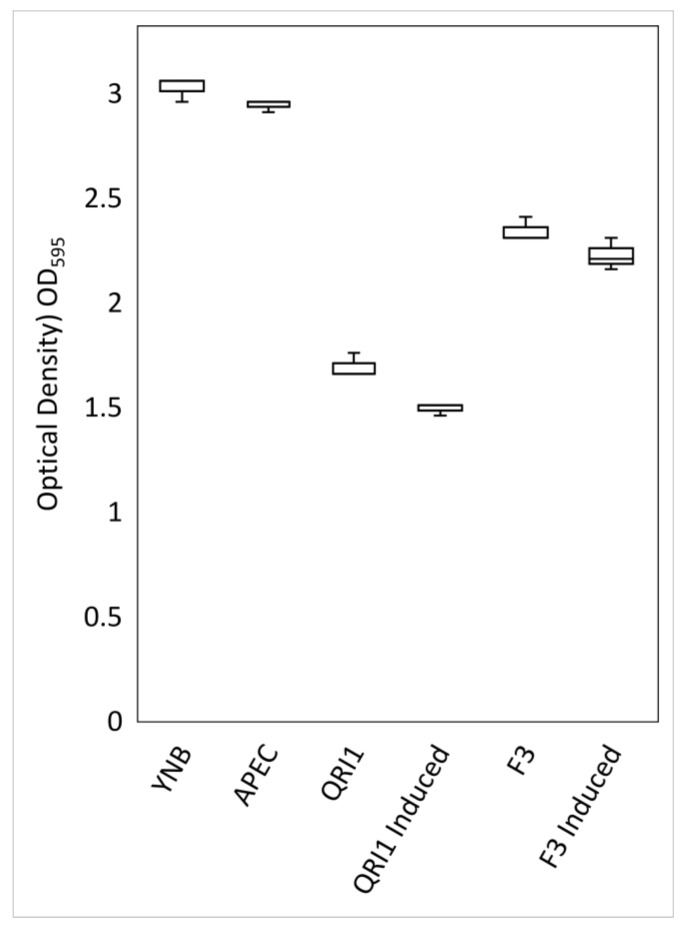
Effect of yeast supernatant on APEC growth. To prepare media, 9 mL of SMB was supplemented with 1 mL of the described supernatant or YNB for control, and APEC was inoculated into each. Optical density values represent measurements after 8 h, and box plots represent data from triplicate cultures.

Supernatants generated for SDS-PAGE also altered bacterial growth when compared to YNB controls. After 8 h of growth, no significant difference was observed in the optical density of SMB + YNB control cultures and SMB + APEC supernatant cultures (Figure 7). However, significant reductions (*p* < 0.001) in optical density were observed in bacterial cultures supplemented with yeast supernatants. Contrary to previous assays, it is perhaps surprising that F3 caused such a reduction in growth rate, but this may have been due to reduced positive effects from secreted amylases when yeasts were not actively growing with bacteria. Encouragingly, a significant reduction (*p* < 0.001) in optical density occurred with the addition of QRI1 supernatants compared to F3, and only in the QRI1 sample was there a significant difference between induced and control supernatants for a strain. These results suggest that, whilst the presence of yeast supernatant resulted in reduced bacterial growth, the degree of this attenuation correlated with the antimicrobial results presented throughout this work. In addition, though this growth rate data only show a reduction of bacterial growth rather than inhibition, the potential loading of antimicrobial peptides within an aliquot of supernatant is likely to be considerably less than when yeasts are actively growing in competition with bacteria. 

## 4. Discussion

The inhibitory activity against avian pathogenic bacteria presented here correlates with different aspects of the previous work describing *M. pulcherrima’s* antifungal properties. In agreement with Sipiczki and Oro, bacterial inhibition for one strain, ICS1, was removed by supplementation of iron into the solid growth medium [22,24,28]. However, this was not evident for the strains QRI1 and DH10, matching the results from Saravanakumar [32]. This outcome supports the hypothesis proposed by Saravanakumar and Gore-Lloyd that *M. pulcherrima* has alternative inhibitory mechanisms in addition to iron sequestration through PA production [21,32]. The data presented here comprise the first example of bacterial induction of secreted proteins in *M. pulcherrima* and supernatants from *M. pulcherrima* liquid cultures significantly reducing bacterial growth in liquid cultures. The antagonistic phenotype of *M. pulcherrima* is likely to be linked to its highly competitive environmental niche of fruits and flowers. Indeed, it is estimated that a quarter of yeast strains isolated from fruits present the killer yeast phenomena, with it being hypothesised that the visitation of insects carrying competing yeasts leads to the development of these antagonistic phenotypes [33]. An enzymatic screen of *Metschnikowia* sp. confirmed the production of several lytic enzymes, including C4 and C8 esterases, valine arylamidase, acid phosphatase and β-glucosidase [34]. Furthermore, the same study concluded that the enzymatic activity of *Metschnikowia* sp. isolated locally was greater than that of strains sourced from culture collection. For *M. pulcherrima* specifically, one isolated strain was shown to secrete cell wall lytic chitinases, production of which was further elevated in the presence of cell wall proteins from a competing fungus [32]. This result is in accordance with the data presented here demonstrating changes in protein secretome when induced by a competitor. Though the bulk of investigation into killer yeast activity has been in relation to other fungi, studies have also demonstrated the antagonistic activity of different yeasts against bacteria. Ullivarri et al. found that cell-free supernatants of *S. cerevisiae* and *Wickerhamomyces anomalus* caused a longer lag phase in the growth of the wine spoilage-associated bacteria *Lactobacillus hilgardii,* though the supernatant volumes added were far greater than those used in this study [35]. Al-Qaysi et al. used zone-of-inhibition screening to demonstrate the antagonistic activity of *Debaryomyces hansenii* against pathogenic bacterial strains including *S. aureus*, *E. coli*, *Klebsiella pneumonia* and *Streptococcus pyogenes* [36]. Combined with these previous results, our findings contribute to the idea that yeasts, and *M. pulcherrima* specifically, use a versatile suite of chemical and physical properties to significantly reduce bacterial growth across multiple environments.

Natural variation among strains of *M. pulcherrima* has previously been shown to generate large phenotypic differences in oleaginous capacity [20,37] and antimicrobial effect. Our methods are partly designed to lower variation by using standardised conditions, media, and growth times. Although our methods are somewhat limited in their ability to capture the complex environments of a host or the heterogeneity of real-world conditions, using our approach with a small sample of 11 strains, we recovered relatively high inhibitory effects, as well as low inhibitory effects. 

## 5. Conclusions

*Metschnikowia pulcherrima* has repeatedly shown high levels of antimicrobial effects across a wide range of environmental conditions and against a broad array of pathogenic microbes. While the most obvious mechanism underlying this antimicrobial activity has always been the production of pulcherrimin, it is equally apparent that other important mechanisms may have more significant roles than this obvious pigment in some environments. Here we found strong evidence for multiple pathways of inhibition, including proteins that are produced specifically in response to the presence of some avian pathogenic bacteria. Critically, we found that in liquid culture environments some isolates and strains of *M. pulcherrima* can have counter-intuitively beneficial effects for some bacteria while suppressing and killing others, and that some of these effects are driven by the secretome of the *M. pulcherrima* strains. How these effects have evolved in natural environments remains an open question, but there are clear routes to exploit these traits in industry. Strains that support a healthy microbiome in agricultural environments and enable better use of novel feed are the most obvious areas for further development. It will be important to uncover how both specific and generic responses in *M. pulcherrima* to ”beneficial” microbes, as well as ”harmful” ones, ultimately shape its role in enabling a stable microbial community. This work would enable the discovery of the molecules that underlie these interactions but also clarify how the regulation and responsiveness in the production of these molecules ultimately determine outcomes.

## Data Availability

Not applicable.

## Supplementary Materials

The following are available online at https://www.mdpi.com/article/10.3390/biology10121227/s1, The full Western Blot can be found in Supplementary Materials.
