# Supplementary material for: The Oleaginous Yeast Metschnikowia pulcherrima Displays Killer Activity against Avian-Derived Pathogenic Bacteria
Source: Biology (Basel). 2021 Nov 24;10(12):1227. doi: 10.3390/biology10121227 (PMC8698481; doi:10.3390/biology10121227)
Supplement: Supplementary file 1 [file biology-10-01227-s001.zip › biology-1410488-supplementary.pdf]

Image in paper – the following slides with the respective original full images of each lane, in order from left to right. I did each of the conditions in triplicate, but I've left the tag over the lane that was used in the below figure (I think for a few of them they're using the same original image, but did it for each just for continuity). Pics were taken with my iPhone.

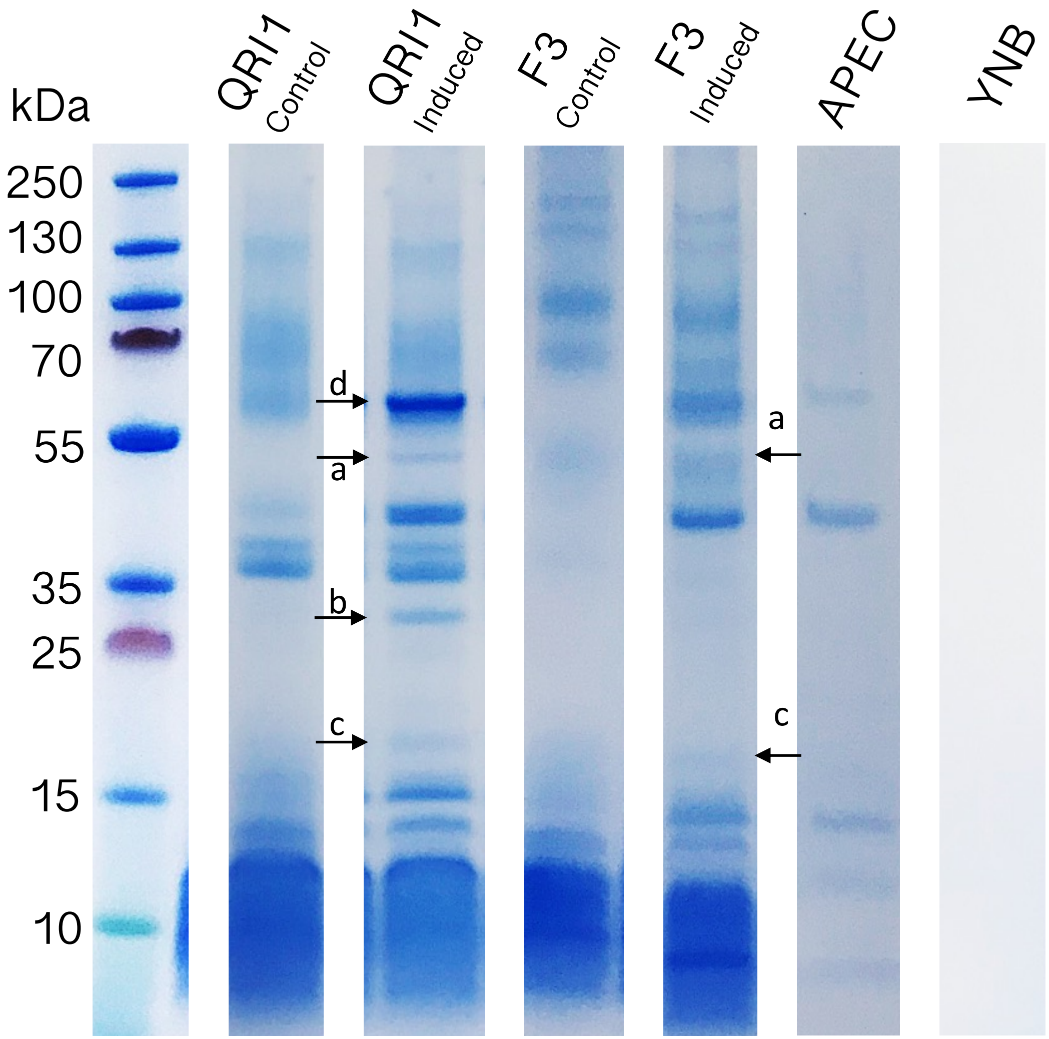

ladder

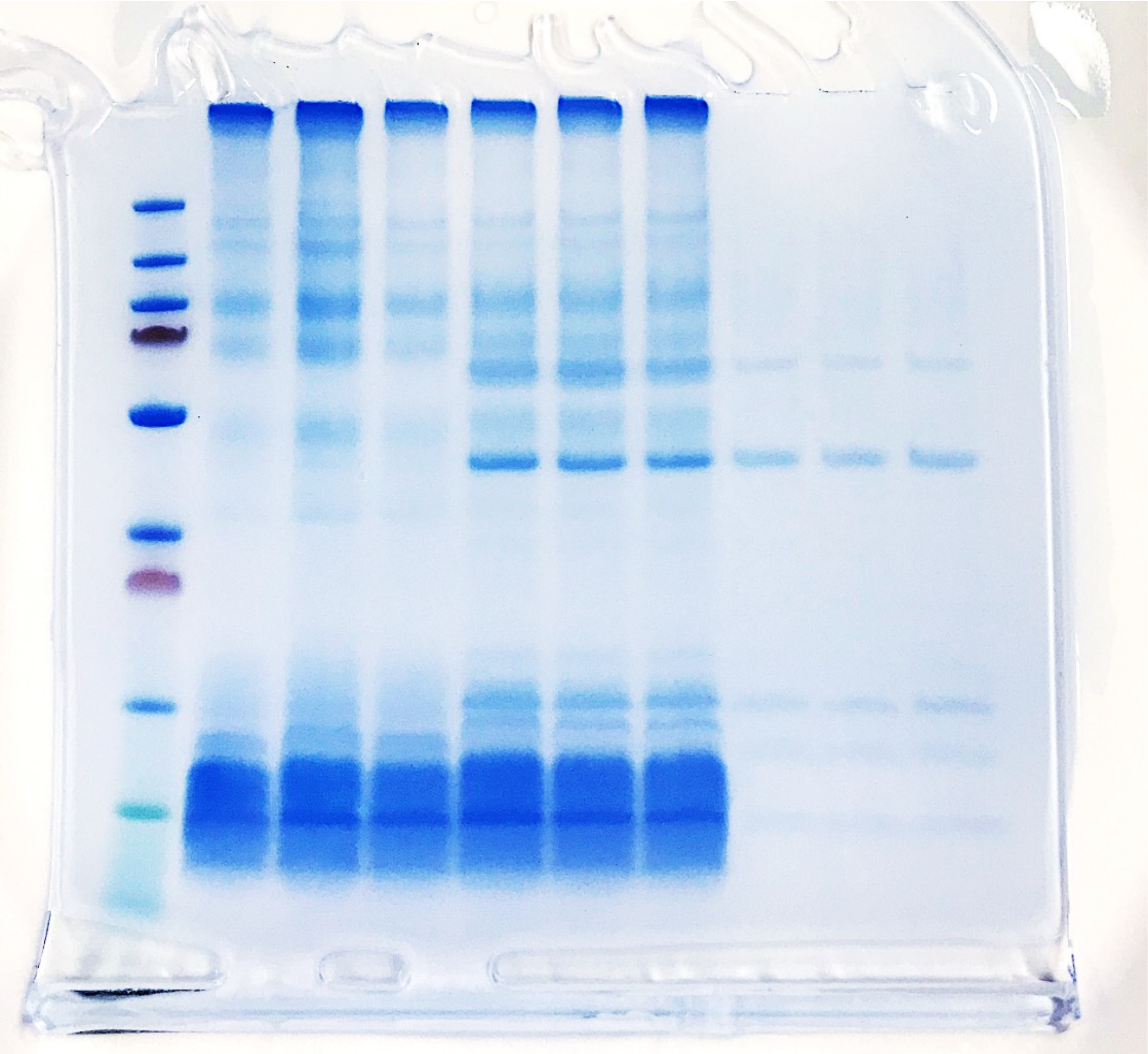

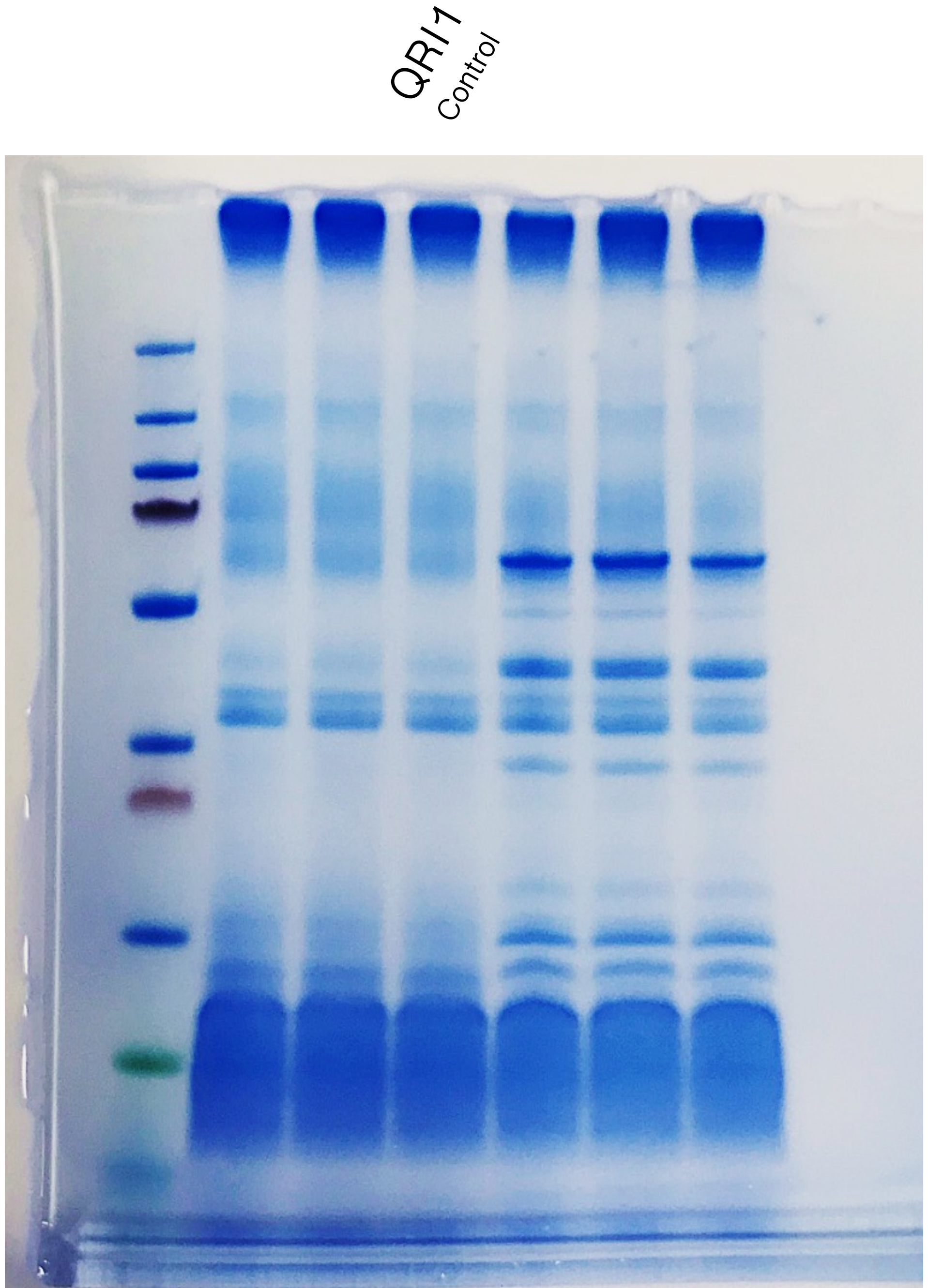
QRI1
Control

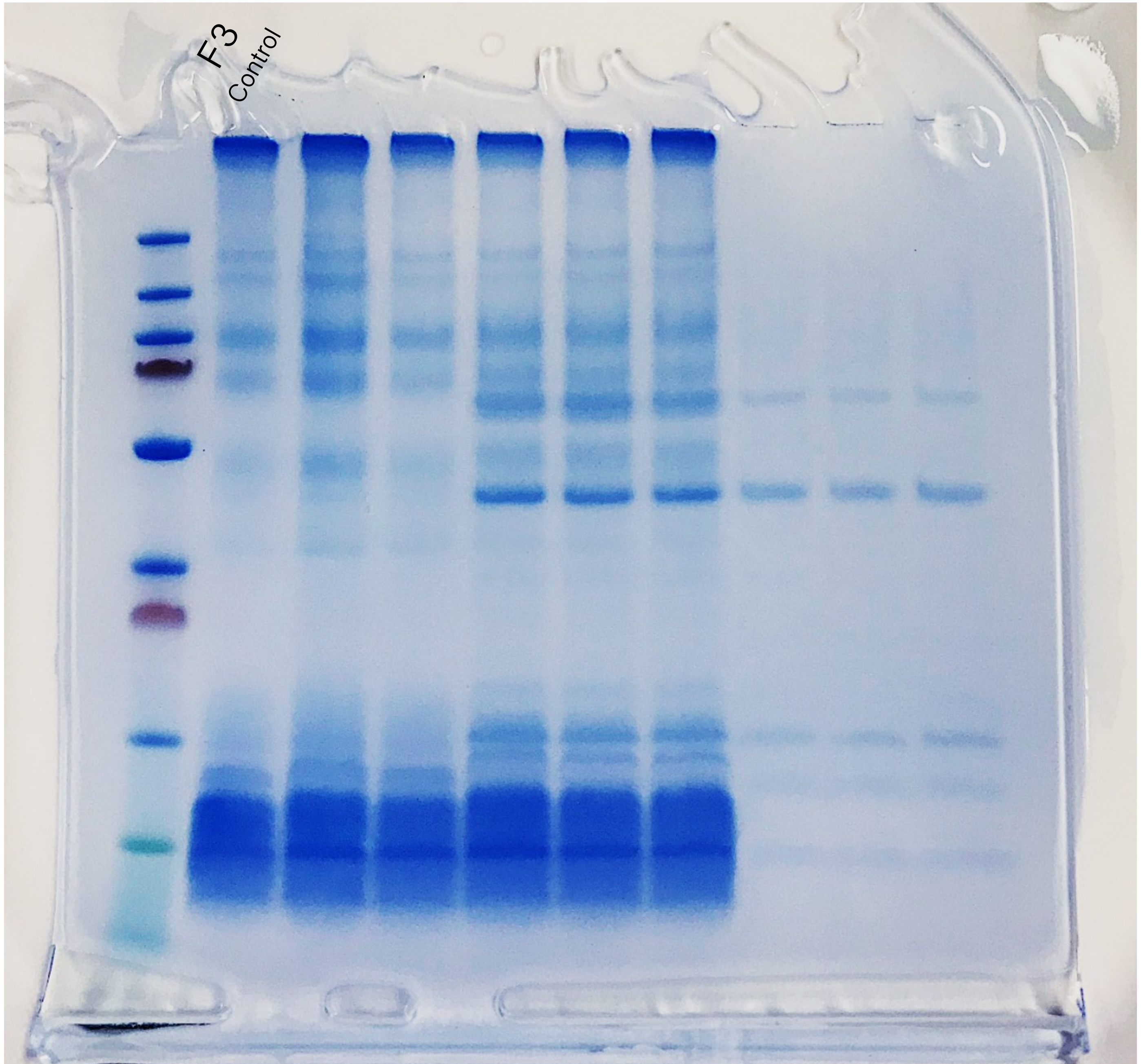
F3
Control

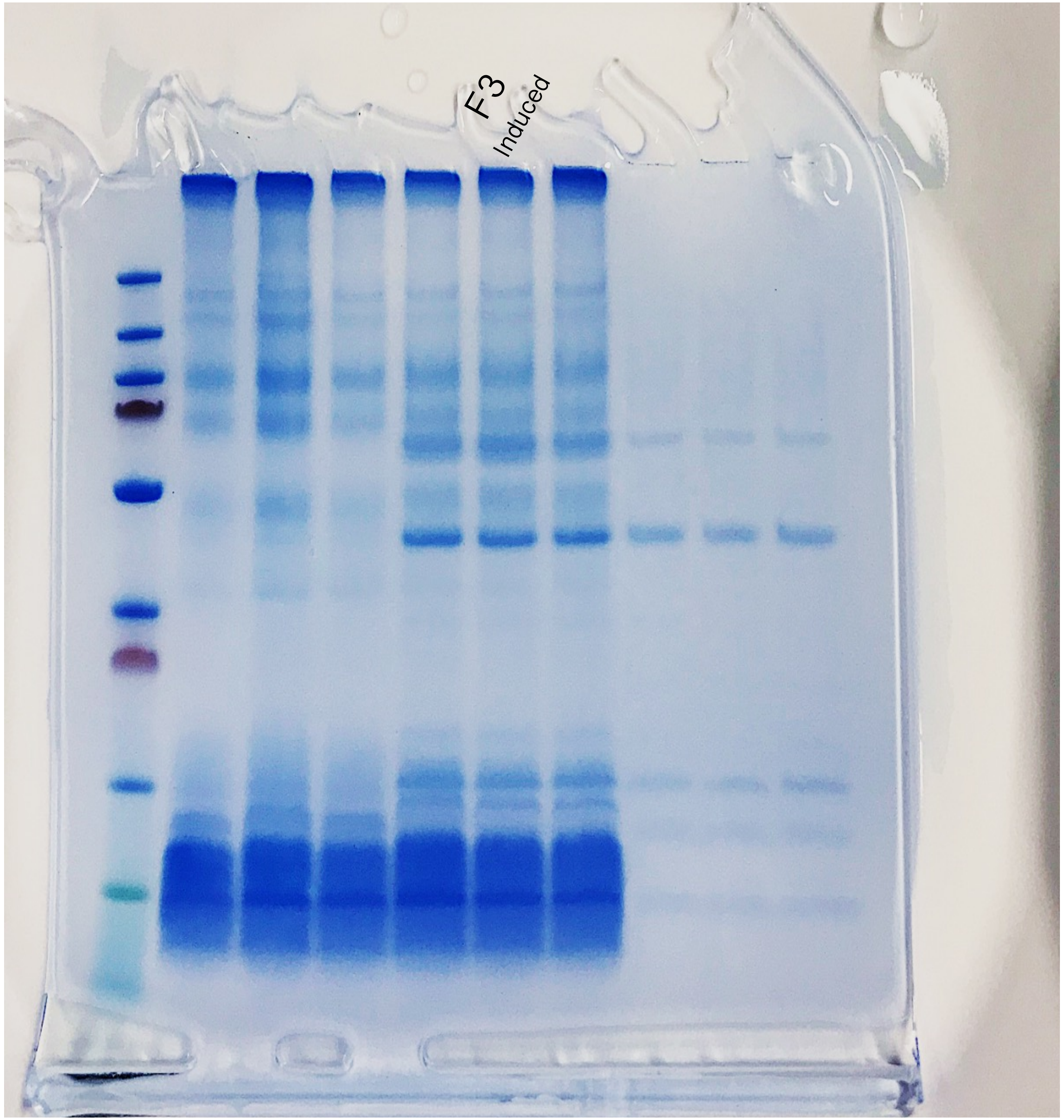

F3
Induced

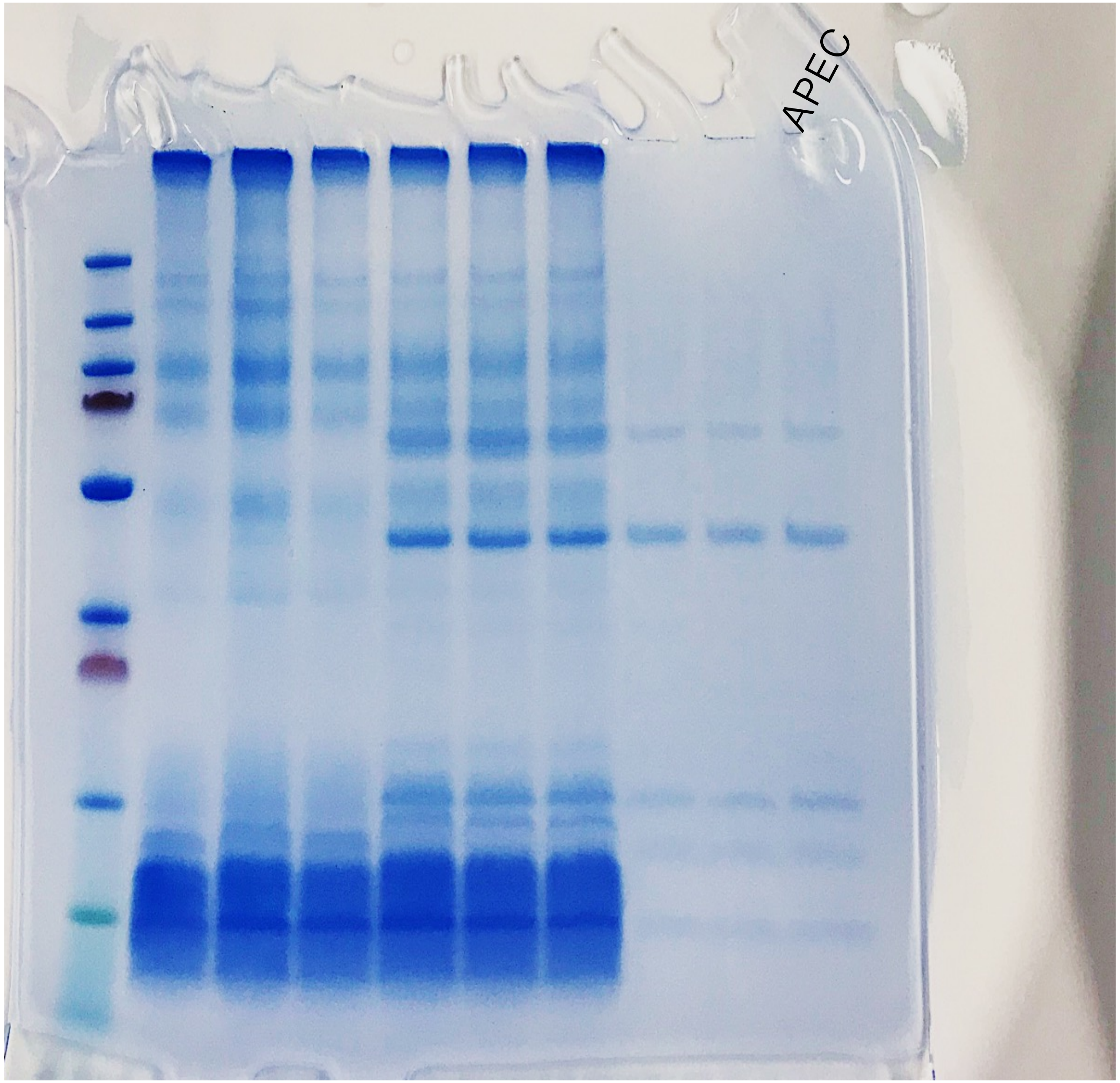
APEC

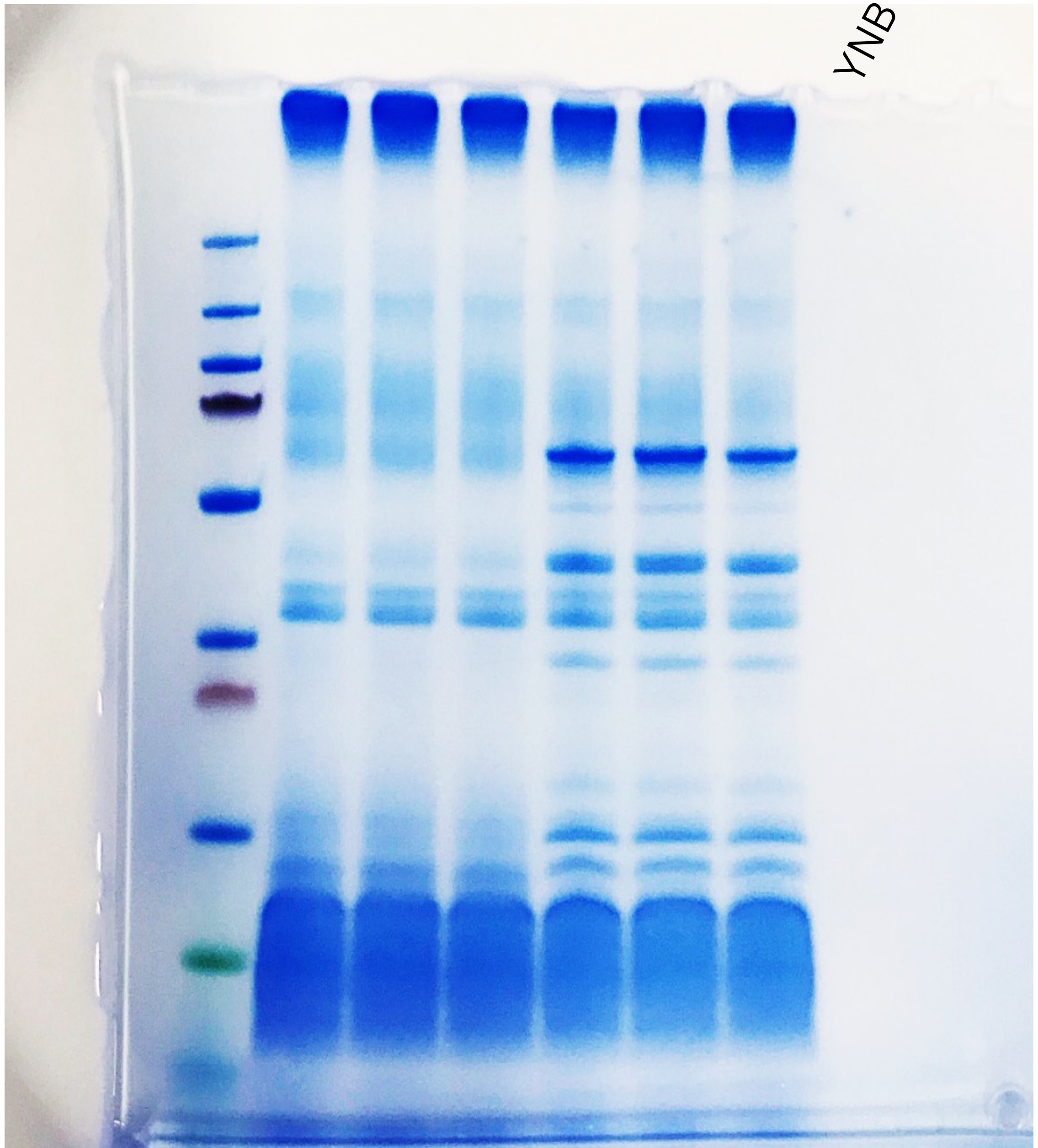
YNB
